# Supplementary material for: Community health worker-delivered counselling for common mental disorders among chronic disease patients in South Africa: a feasibility study
Source: BMJ Open. 2019 Jan 15;9(1):e024277. doi: 10.1136/bmjopen-2018-024277 (PMC6340481; doi:10.1136/bmjopen-2018-024277)
Supplement: Supplementary file 1 [file bmjopen-2018-024277supp001.pdf]

Good Reporting of A Mixed Methods Study (GRAMMS) checklist

**Guideline**	**Section: page**
Describe the justification for using a mixed methods approach to the research question	Methods- under procedures pg. 8
Describe the design in terms of the purpose, priority and sequence of methods	Methods- procedures pg. 7-8
Describe each method in terms of sampling, data collection and analysis	Procedures pg 7-8<br>Analysis: pg. 9-10
Describe where integration has occurred, how it has occurred and who has participated in it	Design: pg. 7-8
Describe any limitation of one method associated with the present of the other method	Discussion pg. 15-17
Describe any insights gained from mixing or integrating methods	Discussion: pg. 15-17

O'Cathain A, Murphy E, Nicholl J. The quality of mixed methods studies in health services research. J Health Serv Res Policy. 2008;13: 92-98.
